# High cumulative doxorubicin dose for advanced soft tissue sarcoma

**DOI:** 10.1186/s12885-020-07663-x

**Published:** 2020-11-23

**Authors:** Zhichao Tian, Yang Yang, Yonghao Yang, Fan Zhang, Po Li, Jiaqiang Wang, Jinpo Yang, Peng Zhang, Weitao Yao, Xin Wang

**Affiliations:** 1 Department of Bone and Soft Tissue, the Affiliated Cancer Hospital of Zhengzhou University and Henan Cancer Hospital, Dongming road, Zhengzhou, 450008 Henan Province China; 2 Huanghe Science and Technology College, Zhengzhou, 450063 Henan Province China; 3 Department of Immunotherapy, The Affiliated Cancer Hospital of Zhengzhou University and Henan Cancer Hospital, Zhengzhou, 450008 Henan Province China; 4 Department of Medical Oncology, the Affiliated Cancer Hospital of Zhengzhou University and Henan Cancer Hospital, Zhengzhou, 450008 Henan Province China

**Keywords:** Soft-tissue sarcoma, Doxorubicin, Dexrazoxane, Cardiotoxicity, Cumulative dose

## Abstract

**Background:**

The recommended cumulative doxorubicin dose in soft tissue sarcoma (STS) treatment was based on cardiotoxicity data from retrospective studies of breast cancer patients. However, the treatment and prognosis of STS and breast cancer are quite different, and reference to breast cancer data alone may not reflect the efficacy of doxorubicin treatment in STS. This study, thus, aimed to review and analyze clinical data of STS patients treated with a high cumulative doxorubicin dose, to provide a reference for treatment selection and clinical trial design.

**Methods:**

We retrospectively collected and analyzed clinical data of patients with advanced STS who received doxorubicin-based chemotherapy from January 2016 to January 2020. The patients were divided into a standard-dose group (who received ≤6 cycles of doxorubicin after the initial diagnosis) and an over-dose group (who were re-administered doxorubicin [doxorubicin-rechallenge] after receiving 6 cycles of doxorubicin therapy discontinuously). Patient characteristics, cumulative doxorubicin dose, objective response rate (ORR), disease control rate (DCR), progression-free survival (PFS), cardiotoxicity incidence, and treatment effectiveness were evaluated in both groups.

**Results:**

A total of 170 patients with advanced STS were recruited (146 in the standard-dose group and 24 in the over-dose group). The average cumulative doxorubicin dose was 364.04 ± 63.81 mg/m2 in the standard-dose group and 714.38 ± 210.09 mg/m2 in the over-dose group. The ORR, DCR, and median PFS were 15.07, 58.9%, and 6 (95% confidence interval [CI]: 5.8–6.5) months in the standard-dose group and 16.67, 66.67%, and 4 (95%CI: 2.0–5.8) months in the over-dose group, respectively. Symptomatic heart failure occurred in five patients (3.42%) of the standard-dose group and in one patient (4.17%) of the over-dose group. In these patients with cardiotoxicity, doxorubicin was discontinued, and all of them died of uncontrolled tumor growth. No drug-related deaths occurred.

**Conclusions:**

The continuation of or rechallenge with doxorubicin beyond the recommended cumulative dose could be a promising therapeutic option in the treatment of chemotherapy-sensitive advanced sarcomas. Further evaluation is necessary in prospective trials.

## Background

As a type of anthracycline, doxorubicin has long been used in chemotherapy for malignancies. At present, doxorubicin is used as a standard chemotherapeutic drug for breast cancer, ovarian cancer, sarcoma, lymphoma, small-cell lung cancer, and many other malignant tumors [1]. Its efficacy in these malignancies has been repeatedly confirmed. However, continuous use of doxorubicin in treatable patients is associated with dose-dependent cardiotoxicity [2]. The initial study of the drug identified a 3% incidence of cardiotoxicity (mainly heart failure) at a cumulative dose of 400 mg/m^2^, 7% at 550 mg/m^2^, and 18% at 700 mg/m^2^ [3]. Another study showed that the incidence of cardiotoxicity in 630 patients treated with doxorubicin was 5.1% at a mean cumulative doxorubicin dose of 283 mg/m^2^ [4]. In other studies, the incidence of doxorubicin-induced cardiotoxicity varied greatly due to the different definitions of cardiotoxicity used [5, 6]. Despite these differences in findings, it is generally accepted that the cardiotoxicity of doxorubicin increases with cumulative dose. Thus, the optimal cumulative dose of doxorubicin remains a key concern in the treatment of many malignancies. The currently accepted safe cumulative dose of doxorubicin is < 450 mg/m^2^ [7–9].

Soft-tissue sarcoma (STS) is a rare malignancy. However, there are still about 40,000 new cases of STS in China every year [10]. About half of these are at the advanced-disease stage or will eventually progress to advanced disease. The first-line treatment for advanced STS is doxorubicin-based chemotherapy [11–13]. However, due to the low incidence of sarcomas, the study of doxorubicin-induced cardiotoxicity is far less common in sarcoma patients than in breast cancer patients. Although the cumulative doxorubicin dose was reported to be significantly higher for sarcoma than for breast cancer, there was no corresponding increase in the incidence of cardiotoxicity in sarcoma [14–16]. In the treatment of STS, the recommended cumulative doxorubicin dose is also 450 mg/m^2^ [13], which is clearly based on data for other malignancies, particularly breast cancer [17]. However, the treatment and prognosis of STS and breast cancer are quite different, and reference to breast cancer data alone may not reflect or improve the efficacy of doxorubicin treatment in STS.

As a major sarcoma treatment center in central China, our institution has used high cumulative doses of doxorubicin in our clinical work to treat a number of patients with advanced STS, because of the lack of other effective treatment modalities [18]. In this study, we aimed to review and analyze the clinical data of these patients to provide a reference for treatment selection and clinical trial design for treating advanced STS.

## Methods

### Patients and eligibility criteria

To provide reference criteria for the selection of a treatment dose and to aid in identifying factors for future clinical trials studying doxorubicin dose in STS, we retrospectively collected and analyzed the clinical data of patients with advanced STS who received doxorubicin-based chemotherapy at the Affiliated Cancer Hospital of Zhengzhou University from January 2016 to January 2020. The inclusion criteria were as follows: (1) patients who were pathologically diagnosed with advanced-stage STS, (2) patients who received at least 2 cycles of doxorubicin-based chemotherapy, and [19] patients with complete clinical and follow-up data.

### Statistical indicators and analysis

According to the cumulative dose and number of cycles of doxorubicin, the patients were divided into two groups: the standard cumulative dose group (standard-dose group) and the over-cumulative dose group (over-dose group). The standard-dose group comprised patients who received ≤6 cycles of doxorubicin after the initial diagnosis, while the over-dose group consisted of patients who were re-administered doxorubicin (doxorubicin rechallenge) after the discontinuation of doxorubicin therapy (6 cycles) as other treatment methods (other chemotherapeutic agents, tyrosine kinase inhibitors or immune checkpoint inhibitors) were ineffective. The effectiveness and cardiotoxicity of doxorubicin therapy in the first treatment phase with 6 cycles (the pre-over-dose group) were also assessed.

The general characteristics, cumulative doxorubicin dose, objective response rate (ORR), disease control rate (DCR), progression-free survival (PFS), and incidence of cardiotoxicity in the two groups were assessed. Treatment effectiveness was evaluated according to the Response Evaluation Criteria in Solid Tumors version 1.1. The baseline therapeutic effectiveness in the over-dose group was based on the target lesion diameter at the beginning of the doxorubicin rechallenge. PFS in the standard-dose group and the pre-over-dose group was defined as the time from the start of doxorubicin treatment to the occurrence of progressive disease or to the switch to another treatment regimen. PFS in the over-dose group was defined as the time from the start of the doxorubicin rechallenge to the occurrence of progressive disease or death. Cardiotoxicity was defined as heart failure with the associated symptoms and clinical signs.

All continuous variables are presented as means and standard deviations, and all categorical variables are expressed as numbers and percentages of patients. Survival analysis was performed using the Kaplan–Meier method with a 95% confidence interval (CI). Figures were drawn using GraphPad Prism 5.0 (GraphPad Software Inc., San Diego, CA, USA). All statistical analyses were performed using SPSS software version 24.0 for Windows (IBM Corp., Armonk, NY, USA).

## Results

### Patient characteristics

A total of 170 patients with advanced STS were recruited. There were 146 patients in the standard-dose group and 24 in the over-dose group. The general characteristics of these patients are shown in Table 1.

**Table 1:** Patient characteristics of standard-dose group and over-dose group

Characteristics	Standard-dose group (*n* = 146)	Over-dose group (*n* = 24)
Gender		
Male	70 (47.95%)	14 (58.33%)
Female	76 (52.05%)	10 (41.67%)
Age	43.30 ± 12.10	38.58 ± 14.01
ECOG PS		
0	71 (48.63%)	7 (29.17%)
1	75 (51.37%)	17 (70.83%)
Histological types		
Undifferentiated sarcoma	36 (24.66%)	6 (25.00%)
Synovial sarcoma	23 (15.75%)	4 (16.67%)
Leiomyosarcoma	20 (13.70%)	3 (12.50%)
Fibrosarcoma	16 (10.96%)	3(10.34%)
Liposarcoma	15 (10.27%)	2 (8.33%)
Angiosarcoma	14 (9.59%)	6 (25.00%)
Epithelioid sarcoma	7 (4.79%)	
MPNST	7 (4.79%)	
Clear cell sarcoma	3 (2.05%)	
Others	5 (3.42%)	
Metastatic or locally unresectable		
Locally unresectable	20 (13.70%)	7 (29.17%)
Metastatic	120 (82.20%)	17 (70.83%)
Both	6 (4.11%)	0
Primary site		
Extremities	101 (69.18%)	14 (58.33%)
Trunk	45 (30.82%)	10 (41.67%)
Metastatic site		
Lung	124 (84.93%)	16 (66.67%)
Other	22 (15.07%)	8 (33.33%)
Mean cycles of doxorubicin chemotherapy	5	11.5

Notes: Data are presented as numbers (percentages) or means ± standard deviations

Abbreviations: *ECOG PS* Eastern Cooperative Oncology Group performance status, *MPNST* malignant peripheral nerve sheath tumor

The youngest patient was aged 17 years, and the oldest was 65 years. The mean age of the standard-dose group was 43.30 ± 12.10 years, while that of the over-dose group was 38.58 ± 14.01 years. Physical fitness was good in both groups, and no patients had an Eastern Cooperative Oncology Group performance status score of ≥2. The most common histopathological subtypes were undifferentiated sarcoma, synovial sarcoma, and leiomyosarcoma in the standard-dose group, and undifferentiated sarcoma, synovial sarcoma, and angiosarcoma in the over-dose group. Most of the patients in both groups had primary lesions in their extremities and lung metastases. Patients in the standard-dose group received an average of 5 cycles of doxorubicin, while those in the over-dose group received an average of 11.5 cycles.

### Treatment and dose modification

**Fig. 1:**
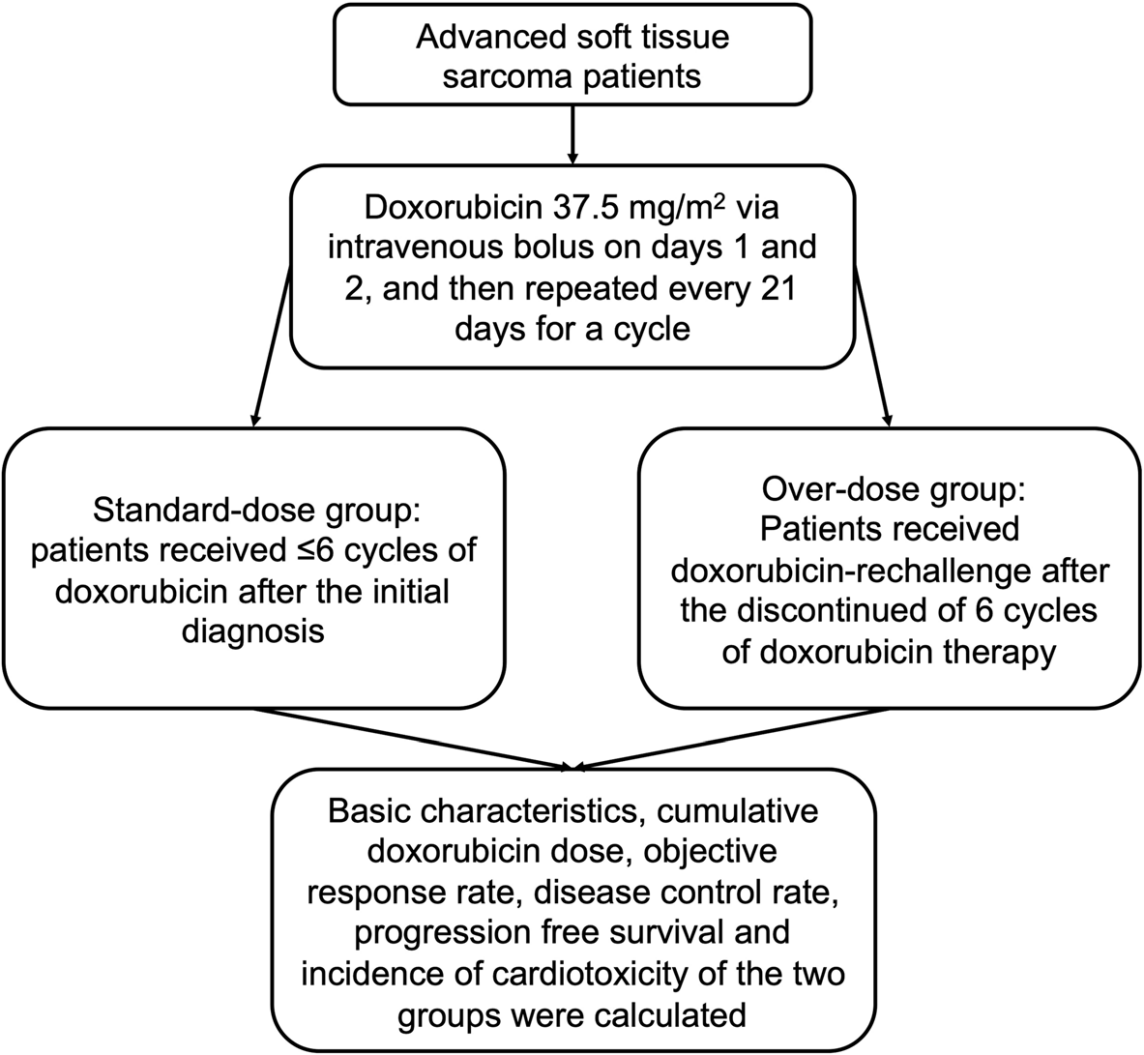
The treatment of patients and the study flowchart used in this study

**Fig. 2:**
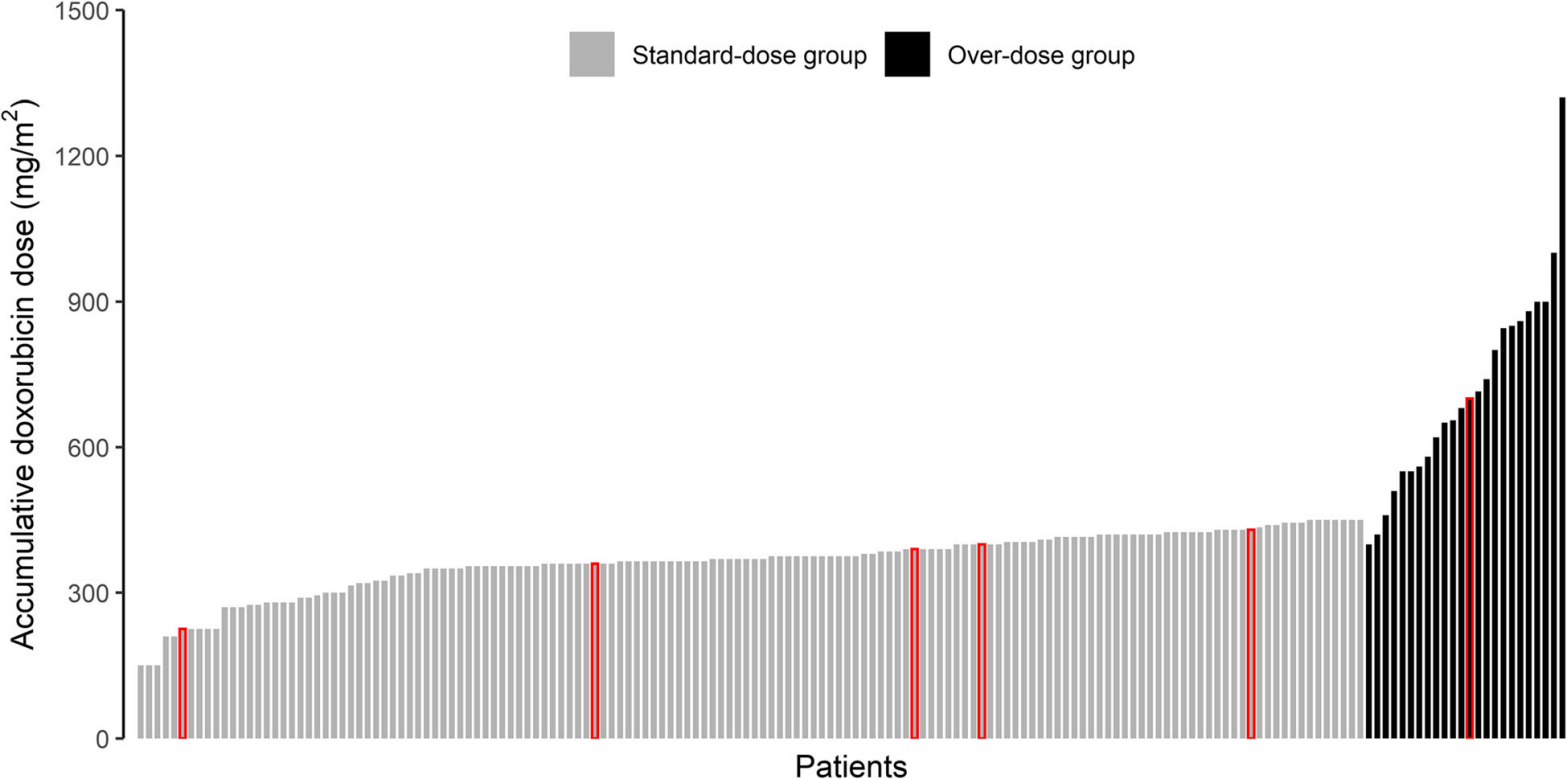
cumulative doxorubicin dose for patients in this study. In red circles are patients who have developed cardiotoxicity

The treatment of patients and the study flowchart used in this study are shown in Fig. 1. All patients received doxorubicin at an initial dose of 37.5 mg/m^2^ via intravenous bolus injection on days 1 and 2 of a 21-day cycle. All patients were also treated with dexrazoxane at the beginning of the doxorubicin treatment. In the standard-dose group, the dose of doxorubicin was modestly reduced in patients with severe adverse events (all of which were hemotoxic), and doxorubicin was administered until disease progression or until 6 cycles were completed. Doxorubicin rechallenge was undertaken in the patients in the over-dose group. In other words, doxorubicin was discontinued in these patients after they received 6 cycles of doxorubicin therapy and then restarted after other treatment methods proved ineffective. The average cumulative doxorubicin dose was 364.04 ± 63.81 mg/m^2^ in the standard-dose group and 714.38 ± 210.09 mg/m^2^ in the over-dose group (Table 2, Fig. 2). The average cumulative doxorubicin dose in the pre-over-dose group was 421.15 ± 47.39 mg/m^2^ (Table 2).

**Table 2:** Clinical characteristics, effectiveness and safety of the two groups

Characteristics	Standard-dose group (*n* = 146)	Pre-over-dose group (*n* = 24)	Over-dose group (*n* = 24)
Accumulative Doxorubicin dose (mg/m^2^)	364.04 ± 63.81	421.15 ± 47.39	714.38 ± 210.09
ORR (%)	15.07%	100%	16.67%
DCR (%)	58.9%	100%	66.67%
Median-PFS (months)	6 (95%CI: 5.8–6.5)	8.15 (95%CI: 5.8–9.5)	4 (95%CI: 2.0–5.8)
Incidence of cardiotoxicity (%)	3.42%	0%	4.17%

Notes: Data are presented as numbers (percentages) or means ± standard deviations

Abbreviations: *ORR* objective response rate, *DCR* disease control rate, *Median-PFS* median progression-free survival

### Effectiveness

**Fig. 3:**
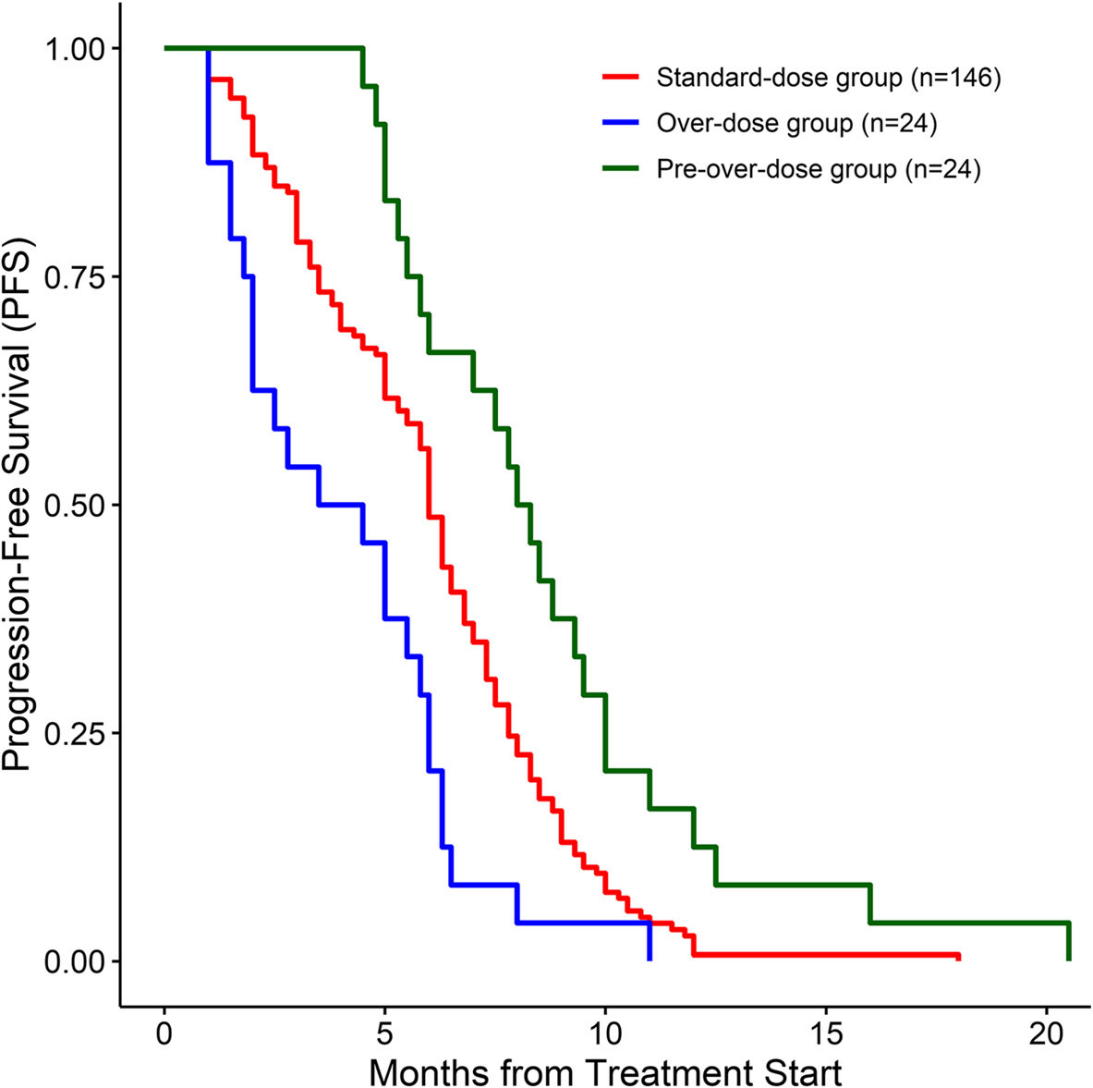
progression-free survival among patients with advanced STS in this study

One patient with angiosarcoma in the over-dose group achieved a complete response. In the standard-dose group, the ORR, DCR, and median PFS were 15.07, 58.9%, and 6 (95% CI: 5.8–6.5) months, respectively (Table 2, Fig. 3). In the pre-overdose group, the ORR, DCR, and median PFS were 100, 100%, and 8.15 (95% CI: 5.8–9.5) months, respectively (Table 2, Fig. 3). In the over-dose group, the ORR, DCR, and median PFS were 16.67, 66.67%, and 4 (95% CI: 2.0–5.8) months, respectively (Table 2, Fig. 3).

### Cardiotoxicity

The incidence of cardiotoxicity in both groups was low (Table 2). Five patients (3.42%) developed symptomatic heart failure in the standard-dose group (Table 2, Fig. 2), whereas only one patient (4.17%) had symptomatic heart failure in the over-dose group (Table 2, Fig. 2). Owing to cardiotoxicity, these patients stopped doxorubicin, and all died of uncontrolled tumor growth. No drug-related deaths were noted in this study.

## Discussion

In this study, we evaluated the ORR, DCR, PFS, and cardiotoxicity of doxorubicin in patients with advanced STS. Patients in the standard-dose group, who received ≤6 cycles of doxorubicin after the initial diagnosis, had an average cumulative doxorubicin dose of 364.04 ± 63.81 mg/m^2^. Patients in the over-dose group had been administered 6 cycles of doxorubicin, and were administered doxorubicin again for various reasons; the average cumulative doxorubicin dose in this group was 714.38 ± 210.09 mg/m^2^. The standard-dose group was assessed in first line, whereas the over-dose group was assessed in further-line (> 1 treatment line). Because of the significant differences in the cumulative dose and timing of doxorubicin and other medication conditions between the groups, as well as the non-randomness of the enrolled cases and the retrospective nature of this study, we did not conduct a statistical comparative test on the two groups of data. Nevertheless, according to the results, in some patients with advanced STS, treatment with an over-cumulative dose of doxorubicin (or rechallenge with doxorubicin beyond the recommended cumulative dose) led to remarkable efficacy without an increase in cardiotoxicity.

Cardiotoxicity in this study was simply defined as the presence of heart failure, with its associated symptoms and clinical signs. There are several definitions of doxorubicin-associated cardiotoxicity, and these definitions are significantly different from each other and based on different incidence rates [4, 20]. With the invention of new detection methods and the improvement of detection sensitivity, researchers have observed cardiotoxicity in low cumulative doses of doxorubicin [21]. A broad definition of cardiotoxicity may make sense for patients with long-term survival, such as breast cancer patients, because of the risk of long-term cardiac disease in these patients [1]. It is clear, however, that for patients with advanced STS with limited survival, the most significant definition of cardiotoxicity would be symptomatic heart failure, as asymptomatic heart damage is of little significance for these patients.

Several studies have proven that a high cumulative dose of doxorubicin is only one of the factors leading to cardiotoxicity. Other factors that can significantly increase the risk of cardiotoxicity include older age (> 65 years), metastasis, infusion regimen, hypertension, overweight, preexisting cardiac disease, concomitant use of other drugs, and pericardial radiotherapy [8, 20, 22, 23]. In this study, there were fewer risk factors for cardiotoxicity in both groups of patients, especially in the over-dose group. Some high-risk patients might have already developed cardiotoxicity during screening, so they were excluded from the over-dose group. Furthermore, we might have unintentionally excluded high-risk patients during patient selection. These could have contributed to the lack of significant increase in the incidence of cardiotoxicity in the over-dose group in this study. Similar to the results of several other studies [8, 24], the cumulative doxorubicin dose in patients with cardiotoxicity in this study was not the highest among the patients, which indicates that the cumulative doxorubicin dose was not the only factor leading to cardiotoxicity.

In addition, dexrazoxane could also be a reason why there was no significant increase in the incidence of cardiotoxicity in the over-dose group in this study. Although dexrazoxane does not completely eliminate doxorubicin cardiotoxicity [21, 25], it is still an option for primary prevention to reduce the risk of doxorubicin cardiotoxicity [7, 25]. Published data have shown that patients with sarcoma can receive a mean cumulative doxorubicin dose of 600-750 mg/m^2^ without a significant increase in cardiotoxicity when they are administered dexrazoxane [15, 16]. Similar to the above studies, this study is also confirmed that dexrazoxane can reduce cardiotoxicity in doxorubicin-treated sarcoma patients.

There are several guidelines regarding the management of doxorubicin-related cardiotoxicity [9, 26]. These guidelines generally provide an upper limit for the cumulative doxorubicin dose based on cardiotoxicity data from retrospective studies of breast cancer patients, but are limited in that they do not consider some specific patients. In these specific patients, the benefits of a high cumulative doxorubicin dose may outweigh its costs. The results of this study suggest that such ideas and guidelines should be reviewed and revised. The cumulative dose of doxorubicin is a factor that needs to be considered in individualized treatment, but it should not be a limiting factor. Patients with advanced STS have fewer effective treatments and a shorter survival than patients with breast cancer. Doses above the standard upper limit of the cumulative dose of doxorubicin at a certain stage (when no other effective treatment is available) should be administered in patients with advanced STS who are in a good physical condition or otherwise, if doxorubicin remains effective.

Although the small sample size and the retrospective design of this study affect the credibility of the results, the results obtained suggest that the continuation or rechallenge with doxorubicin beyond the recommended cumulative dose is a promising option to improve survival in patients with advanced STS. Nevertheless, further evaluation through prospective trials is necessary to validate these findings. Early monitoring of doxorubicin-related cardiotoxicity is also worthy of further study.

## Conclusions

A high cumulative dose of doxorubicin for advanced STS was associated with a better efficacy. Moreover, there was no significant increase in cardiotoxicity. The continuation or rechallenge with doxorubicin beyond the recommended cumulative dose could be a promising therapeutic option in the treatment of chemotherapy-sensitive advanced STS. Further evaluation in prospective trials is therefore warranted.

## Data Availability

The datasets used and/or analysed during the current study are available from the corresponding author on reasonable request.

## Abbreviations

CI: Confidence interval
DCR: Disease control rate
ECOG PS: Eastern Cooperative Oncology Group performance status
MPNST: Malignant peripheral nerve sheath tumor
ORR: Objective response rate
PFS: Progression-free survival
STS: Soft tissue sarcoma
